# Collaborative action for person-centred coordinated care (P3C): an approach to support the development of a comprehensive system-wide solution to fragmented care

**DOI:** 10.1186/s12961-017-0263-z

**Published:** 2017-11-22

**Authors:** Helen M. Lloyd, Mark Pearson, Rod Sheaff, Sheena Asthana, Hannah Wheat, Thava Priya Sugavanam, Nicky Britten, Jose Valderas, Michael Bainbridge, Louise Witts, Debra Westlake, Jane Horrell, Richard Byng

**Affiliations:** 1 Community and Primary Care Research Group, Clinical Trails and Population Studies, Peninsula School of Medicine and Dentistry, Room N14, ITTC Building, Plymouth Science Park, Derriford, Plymouth, Devon PL6 8BX United Kingdom; 2 School of Law, Criminology and Government, University of Plymouth, Portland Villas, Plymouth, Devon PL4 8AA United Kingdom; 3 NIHR CLAHRC South West Peninsula (PenCLAHRC), Institute of Health Research, University of Exeter Medical School, St Luke’s Campus, Heavitree Road, Exeter, EX1 2LU United Kingdom; 4 Health Services & Policy Research, University of Exeter Collaboration for Academic Primary Care, APEx, University of Exeter Medical School, St Luke’s Campus, Heavitree Road, Exeter, EX1 2LU United Kingdom; 5 Primary Care Development Somerset Clinical Commissioning Group, Working Together to Improve Health and Wellbeing, Wynford House, Lufton Way, Yeovil, Somerset BA22 8HR United Kingdom; 6 South West Academic Health Science Network, Pynes Hill Court, Pynes Hill, Exeter, EX2 5AZ United Kingdom

**Keywords:** Person-centred coordinated care, Collaborative action, Integration

## Abstract

**Background:**

Fragmented care results in poor outcomes for individuals with complexity of need. Person-centred coordinated care (P3C) is perceived to be a potential solution, but an absence of accessible evidence and the lack of a scalable ‘blue print’ mean that services are ‘experimenting’ with new models of care with little guidance and support. This paper presents an approach to the implementation of P3C using collaborative action, providing examples of early developments across this programme of work, the core aim of which is to accelerate the spread and adoption of P3C in United Kingdom primary care settings.

**Methods:**

Two centrally funded United Kingdom organisations (South West Collaboration for Leadership in Applied Health Research and Care and South West Academic Health Science Network) are leading this initiative to narrow the gap between research and practice in this urgent area of improvement through a programme of service change, evaluation and research. Multi-stakeholder engagement and co-design are core to the approach. A whole system measurement framework combines outcomes of importance to patients, practitioners and health organisations. Iterative and multi-level feedback helps to shape service change while collecting practice-based data to generate implementation knowledge for the delivery of P3C. The role of the research team is proving vital to support informed change and challenge organisational practice. The bidirectional flow of knowledge and evidence relies on the transitional positioning of researchers and research organisations.

**Results:**

Extensive engagement and embedded researchers have led to strong collaborations across the region. Practice is beginning to show signs of change and data flow and exchange is taking place. However, working in this way is not without its challenges; progress has been slow in the development of a linked data set to allow us to assess impact innovations from a cost perspective. Trust is vital, takes time to establish and is dependent on the exchange of services and interactions. If collaborative action can foster P3C it will require sustained commitment from both research and practice. This approach is a radical departure from how policy, research and practice traditionally work, but one that we argue is now necessary to deal with the most complex health and social problems.

## Background – the problem of fragmented health and social care

Fragmented and poorly coordinated care are enduring problems for health and social care systems worldwide [1]. These problems impact most acutely on individuals with complex bio-psychosocial needs [2–8], older individuals considered as ‘frail’, and those with multiple long-term conditions [9–11]. Poor coordination occurs most often when care spans the health service and non-statutory and social care boundaries, but is not limited to those interfaces [12]. Within healthcare in England (e.g. general practice, community nursing, mental health services and acute hospitals), there is also a failure to ensure key clinical functions for the individual patient are delivered in a coordinated and person-centred manner. These include preventing and responding to urgent care needs, rational management of multiple long-term conditions (polypharmacy, self-care) and support to promote social health along with mental and physical wellbeing. Furthermore, the burden of care for these individuals is high [13–15] in emotional, practical and financial terms, and impacts upon practitioner morale and patient outcomes [16, 17]. This paper describes a comprehensive approach to address the enduring problems of non-person-centred and fragmented care, by uniting researchers, professionals, patients and a range of delivery organisations to collectively address this problem.

In the United Kingdom, three potent and interacting problems have contributed to the fragmentation of health and social care over the last 25 years. The first resides in the increasing specialisation of medicine and professional roles, and the second in governments’ initiation of repeated, rapid cycles of service reorganisation, privatisation and contracting [18]. The third problem, and the one addressed through our programme of collaborative action, concerns the nature of the available evidence and the accessibility of it to inform service delivery improvements. This paper details an innovative approach to knowledge mobilisation that we are using which combines evidence from research, knowledge from practice, and information from routinely collected data to flow around and be used within complex health and social care systems.

Current evidence concerning how to implement integrated care is hard to use in a meaningful way because of its disparate nature and the mismatch between long research cycles and the needs of service redesign. In addition, confusion between different but related concepts such as integration, care coordination and continuity of care [19], add further challenges. For example, ‘integrated care’ is related to previous ‘solutions’, such as shared care, chronic disease management and collaborative care [20–22], and was for a period focussed in health and social care, but has now also been deflected for quite different purposes such as cost containment [23, 24] and the vertical integration of care (i.e. the coordination of acute and community services by a single provider or a linked set of providers) [19].

Policy-makers and commissioners perceive this all-embracing concept of ‘integration’, with its focus on removing discontinuities in care, as the solution to fragmented care. The problem here is that ‘integration’ is a diverse concept with numerous definitions and a multitude of interacting dimensions and interdependencies across a system [25]. Such complexity is not easily nor simply translated into implementation models, and with context often ignored, efforts towards implementation are often hindered despite energised initiatives (cf. United Kingdom Integration Pilots and Pioneers) [26].

Drawing upon organisational theory and empirical research, Leutz [27] and Sheaff [3] avoid the temptation of giving the term ‘integration’ a moral loading, defining the integrated organisation of care as:

“*A form of organisation that contains a wide range of services (above all, primary medical care) and by coordinating them attempts to produce the continuities of care (cross-sectional, longitudinal, flexible, relational, and informational) through pooling the funds and resources for the different areas of its work, enabling it to provide whichever services it judges the most suited to the patient and most economical overall, irrespective of the received division of labour and without concern for the internal distribution of costs*.”

Correspondingly, networks of organisations could be described as ‘integrated’ to the extent that they resemble this description. Notwithstanding the above definition, a model or ‘blue print’ for integrated care has yet to be established in terms of the necessary core features. The policy literature, although high on aspirations, also fails to provide details about how to implement core changes [28–30]. Given the lack of coherent guidance, it is unsurprising that healthcare systems have failed to significantly reduce fragmentation [31–35] and improve outcomes for those with complex healthcare needs [16, 36, 37].

A further challenge for services attempting integrated initiatives is that many models have not been sufficiently and robustly tested, at least in the United Kingdom. This is partly a result of the fracture between general practice and community health services, which is built into the present architecture of the NHS. Indeed, it is one of the two big features that NHS structural ‘reforms’ have left practically untouched (the other being NHS funding through taxation). For example, while policy prompted reconfigurations of hospital and community trusts can be imposed by internal management processes, it is harder to bring together needs-rated social care with tax-based NHS care, and to integrate multiple general practices (small businesses) with NHS bureaucratic trusts. Recent National Health Service England policy, outlined in the General Practice Forward View [38] and the Five Year Forward View [39], aspires to unify services by linking such service divisions. However, within the current United Kingdom context of purchaser provider splits and cumbersome contractual frameworks, it is still hard to envisage where and how these bridges will be built. It is unsurprising, therefore, that the most recent experiments with ‘integrated care’ (e.g. Integrated Care Pioneers) in the United Kingdom have failed to meet expectations to address the problems faced by patients [26, 40].

The following sections describe an approach to knowledge mobilisation being tested in the south west of England to generate and share knowledge and evidence to support the implementation of a comprehensive model of person-centred coordinated care (P3C) [41].

## The generation of accessible and timely knowledge through collaborative action to support the implementation of P3C

### General approach

Health systems are responding actively to policy imperatives for ‘integration’ and person-centred care, but these efforts are commonly piecemeal, untested, unevaluated and carried out in isolation, often in response to local leaders or crises. In the United Kingdom, whole system redesign has also begun to gain traction and, with this, the recognition that ‘culture’ change towards more integrated or coordinated care, which is also person centred, requires support and energy spanning different organisational levels, i.e. from individual practitioner to multi-professional team to whole provider-organisation, commissioner (payers) and beyond [5, 42]. Without concurrent and rigorous formative evaluation [43], the learning from these innovations is often short lived or ignored. Furthermore, with little understanding of how the context influences implementation, opportunities to create a wealth of insight are often lost. Consequently, ‘whole system’ implementation knowledge is well timed and requires the appropriate melding of traditional approaches to research (e.g. large randomised controlled trials, etc.) with those that can flex and explore the role of context (e.g. quality improvement, realist evaluation, action research). In spite of this, there are few comprehensive programmes that aim to (1) support the implementation of evidence-informed practice (where it exists) and (2) develop practice-based evidence about what works.

We have set about bringing together a ‘whole systems’ realist evaluation [44] incorporating service redesign, implementation, education, evaluation and research. We are using co-designed ‘Collaborative Action’ [45] in the context of the South West Peninsula Collaboration for Applied Health Research and Care (SW CLAHRC). Our approach shares much with that of Glasgow et al.’s [46] ‘Evidence Integration Triangle’, namely, prioritising actionable feedback to practice, a participatory implementation process, the shared understanding by participants of key components, and practical and ongoing measurement within a multilevel context. We hope that this region-wide initiative of collaborative groups (researchers, commissioners, providers and practitioners) will continue to bring a clarity of purpose and continued use of our common evaluation framework. Through this supportive process we are putting P3C into practice locally whilst also providing a receptive context to support more research, thus contributing to a coherent body of knowledge.

### Support for local initiatives: measurement, theory and learning from experiential and other evidence

The Five Year Forward View developed by NHS England presents a radical vision of reformed and integrated public services working alongside communities, social networks and the voluntary sector to support people living with long-term conditions [39]. A number of national programmes with local initiatives (Integrated Personal Commissioning programme [47], Vanguards [48], Realising the Value [49]) aim to embed person- and community-centred approaches to deliver this vision. To support these local initiatives, we have developed a taxonomic framework for P3C [41, 50, 51], and tools to monitor and develop this at a practice level [41, 51]. This framework has been developed from our multidimensional definition of P3C, which is presented in Table 1.

**Table 1:** The extended South West Peninsula Collaboration for Applied Health Research and Care definition of person-centred coordinated care (P3C)

Person-centred care	The co-creation of care between the patient, their family and informal carers, and health professionals. This definition is becoming widely used by many international organisations and WHO [58–61], and has been translated into a proven approach and used at the Gothenburg University Centre for Person Centred Care. Person-centred care strives to see an individual as bio-psycho-social whole, as a person and not a disease or a collection of conditions.
Capabilities and resources of the person and their wider social context	Psycho-social and environmental resources that are non-clinical and have a community focus. This is commonly being referred to as ‘community-centred approaches’ that complement other types ofinterventions that focus more on individual care and behaviour change, or on developing sustainable environments. These approaches acknowledge the importance of social capital for health and wellbeingto flourish, and acknowledging people as having capabilities and resources [62].
Coordinated care	Care coordination is the deliberate combining, in the necessary forms and sequence, of patient care activities by three or more participants (including the patient) so as to deliver the healthcare chosen for the patient [63]. From a person or family perspective, care coordination is any co-operative activity that helps ensure that the individual’s needs and preferences for health services are met, with effective information sharing across people, work-groups, organisations, and sites over time [63].

**Fig. 1:**
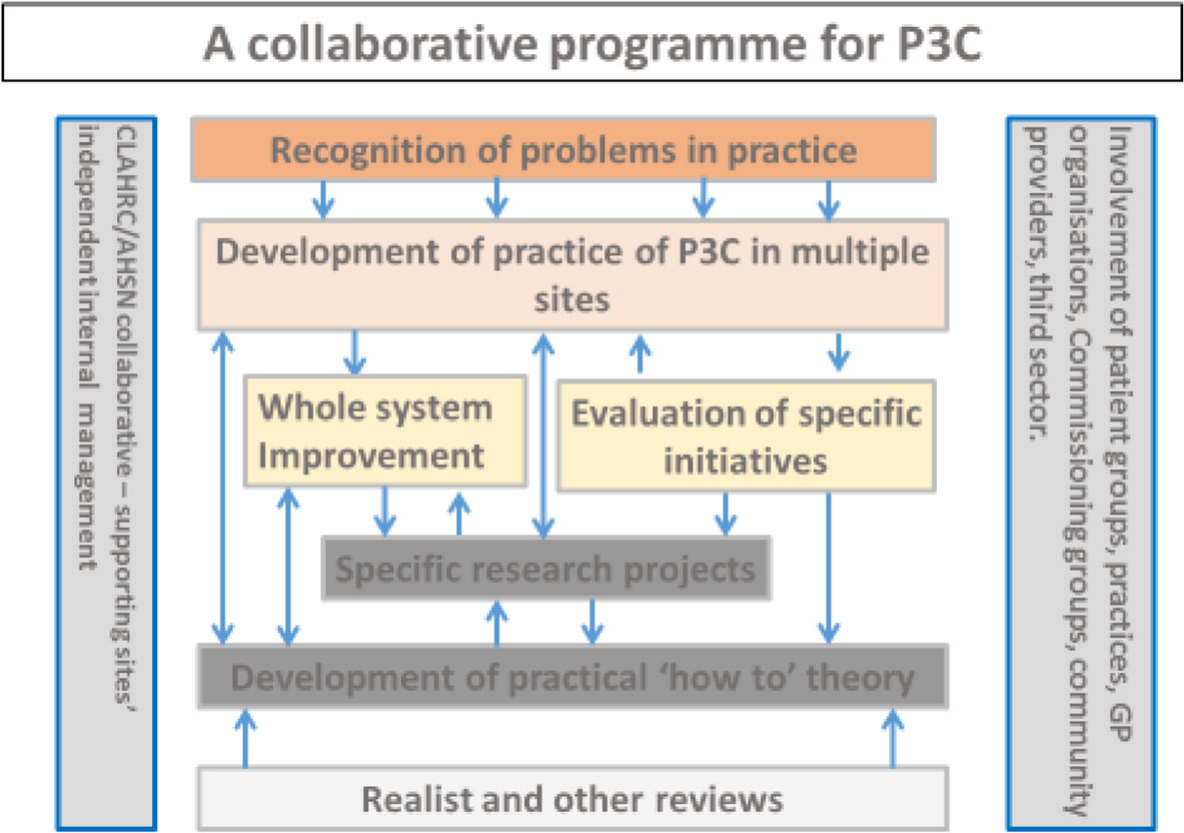
Collective action: an alignment of resources for promoting and supporting person-centred coordinated care (P3C). Grey vertical pillars represent the positioning and type of organisations that are partners in the Collaborative effort. The light grey box and orange box depict the ways in which we come to know of the challenges and potential solutions to service redesign for P3C, and how we use this knowledge to inform practice and our emerging theory. The dark grey boxes and the beige box represent how, through specific projects and service development innovations, we are able to develop insights about what works and how we feed this back into practical efforts to support on-going development. The yellow boxes represent the scale of change, this could be a specific service or a system wide approach and how knowledge from these initiatives flows into the development of practice, the development of theory or defining specific research projects. The blue arrows represent the flow of knowledge around the system

Using ‘Collaborative Action’ [45] and our evaluation framework, we are supporting the implementation of P3C. This involves expert practitioners, managers and researchers working together to help ensure progress in service provision by investigating how, where and why models work. This strategy presupposes that valuable knowledge can be derived from the evaluation of innovations. It also entails accepting that, while we can inform practice by what we do know (using evidence where we have it), we (researchers, practitioners, managers, patients and their carers) also acknowledge what we do not know, and are ready to try things out and create opportunities for experiential learning (including formalising already existing tacit or undocumented knowledge). This ‘Collaborative Action’ is depicted in Fig. 1 (where the arrows represent a flow and exchange of different types of knowledge or activities).

**Fig. 2:**
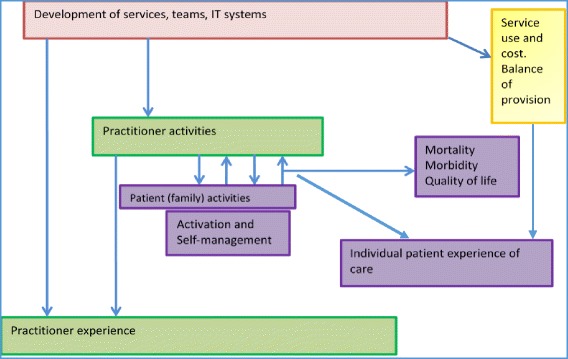
Overarching logic model and evaluation domains for person-centred coordinated care across a system. Pink box represents the organisational changes and support that needs to take place with the arrow linking to the potential impact of this on how practitioners work with patients and how this impacts on their experiences of delivering care. The centre purple boxes represent patient and family/supporter activities and how these influence and are influenced by care interactions. The large blue arrows show how these activities have the potential to influence patient outcomes and experiences of care. The yellow box represent system outcomes and processes around cost and provision of care. These processes and outcomes are influenced by organisational processes (pink box) and, in turn, influence patient experiences of care

This collaborative approach facilitates the transfer and synthesis of different types of knowledge (i.e. published evidence and that developed from local practice) between specific local and wider national/international settings. Generating knowledge this way has the potential to make research more relevant to local ways of delivering care, i.e. by generating more practice-based evidence [52]. These strands are then synthesised and fed back into an emerging understanding of what P3C is and how to support its implementation. This knowledge can also help develop an overarching causal framework (programme logic) to track how changes (e.g. team working, practitioner-patient interactions, etc.) occur and how they relate to improvements in outcomes (Fig. 2).

### A consistent evaluation framework for P3C

Generating useful knowledge and improving practice models is facilitated by the use of a similar evaluation framework across settings. Based, therefore, on the changes anticipated in the overarching programme logic (Fig. 2), we are using a combination of co-selected metrics across core domains of interest (e.g. practitioner and patient experience [51, 53], patient activation [54], patient well-being [55], morbidity, mortality, cost and organisational processes [41]). Local services benefit from the results of the evaluation as these are used to shape the ongoing development of the model. We have found that supporting the development of specific evaluation frameworks for projects based on their objectives and targets improves quality, competency and ownership. Supporting health economies to develop the core measurements (e.g. cost of care, admission rates, experience of care, patient reported outcomes) and embed ways to collect data is helpful to monitor and understand the whole system. This will help create the knowledge that we need about how local care providers produce – or fail to – continuity of care for people with multiple chronic health problems. Crucially for P3C, this approach helps identify the mechanisms that explain how causal relationships occur (through the interaction of people’s reasoning and the resources available to them) [56, 57].

**Fig. 3:**
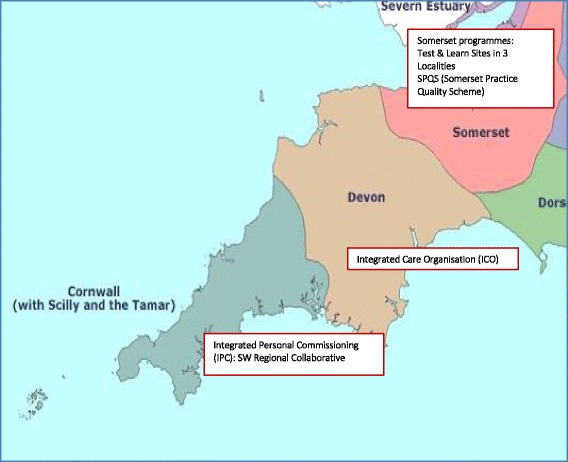
South West United Kingdom practice-based evaluations of person-centred coordinated care. This figure depicts a map of the South West of England showing the counties and the sites with which the collaborative works

**Fig. 4:**
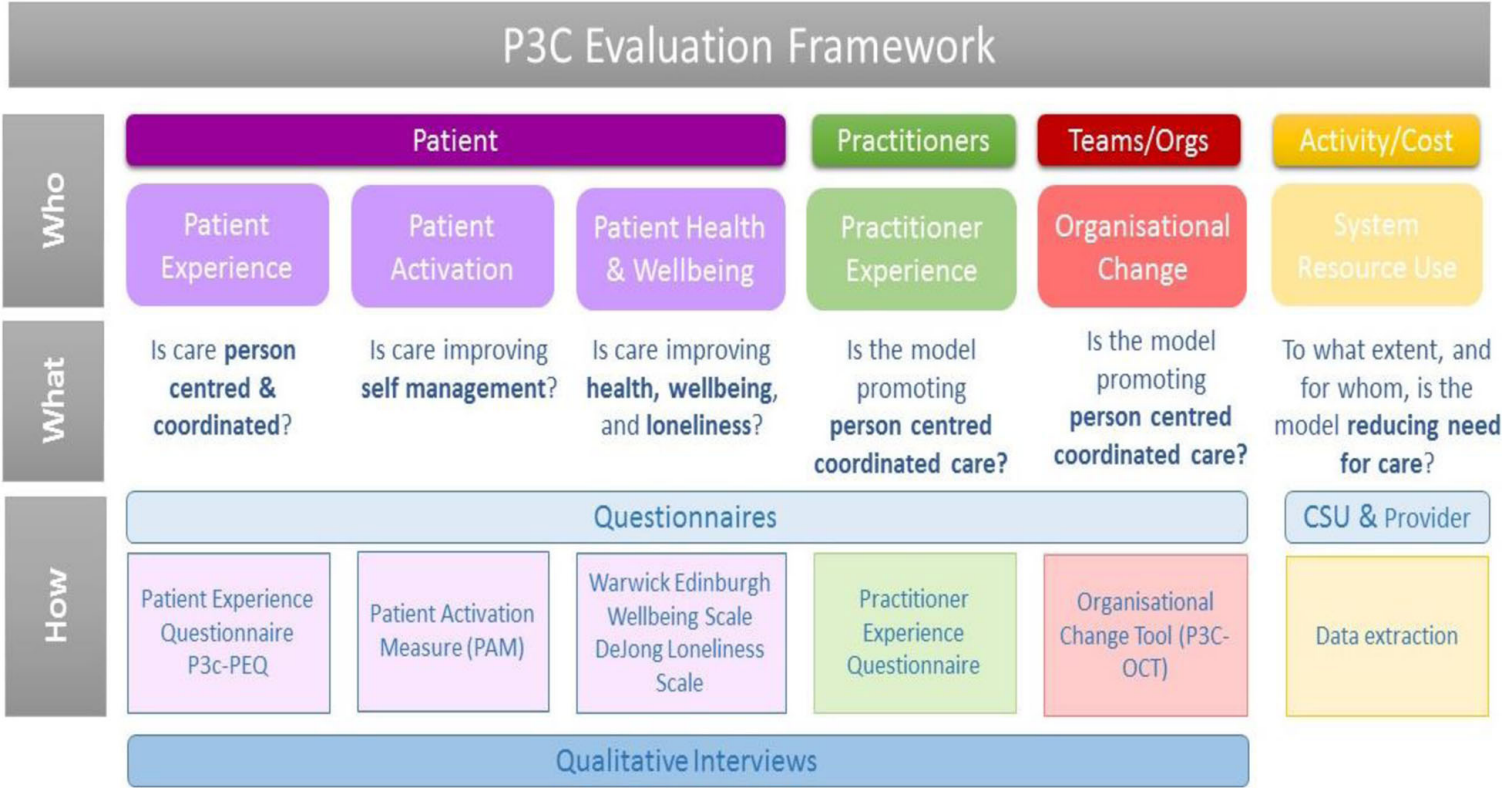
Multi-perspective, multi-level measurement of change with specified measures. Purple boxes depict patient outcome domains, related questions and the measures used to gather this data. Similarly, the green boxes show the domains of interest aimed at practitioners. The red boxes depict the organisational process domains and the yellow boxes describe the cost activity outcomes to be measured. *CSU* clinical support unit

Over the past 2 years, our collaborative effort has involved engagement and working with diverse local sites (Fig. 3) using a consistent multi-level and multi-perspective evaluation framework (Fig. 4) for the evaluation of specific P3C innovations and system change. This framework reflects the complex interventions and organisational level changes that occur during P3C service reconfigurations. More importantly, however, it captures and uses the voice of patients and professionals to shape redesign efforts. For this purpose, we also collect multi-perspective data at both individual and group level (Fig. 4).

This evaluation framework assesses if service change and developments are achieving pre-specified outcomes towards P3C. Using a three tiered and multi-perspective longitudinal mixed methods approach, the framework encompasses qualitative data, questionnaire data and service use and data (Fig. 4). Service use data capture allows for the assessment of patterns of service contact (GP attendance, hospital admissions, etc.) following exposure to an intervention with the intention to derive cost calculations. Staff experience data provides an insight into their perspective, whilst individual patients’ perspectives are sought to assess if care has improved or worsened [51], and if there are any perceived improvements in health, wellbeing, loneliness and self-management. We have included loneliness and self-management as measurement dimensions as these were hypothesised as important to the target population of older people at risk of isolation with long-term conditions. This framework is being used in the English counties of Somerset and Devon (Fig. 3) and was co-designed with local system commissioners and providers in each of the three Clinical Commissioning Groups. This is being used both to monitor the system as a whole over time, and to evaluate particular initiatives and innovations. Generalisable knowledge is derived firstly through reviewing literature, using realist synthesis where feasible, and by comparison of data across the evaluation sites focusing on practitioner-patient interaction and organisational processes. We are also examining the process of implementation, both through the lens of existing knowledge about changing practice and quality improvement in the NHS, and also identifying specific organisational constraints and facilitators for developing system-wide P3C.

## Progress so far

Over the past 2.5 years, extensive efforts have been made to foster partnerships between researchers and health and social care organisations at various levels. This has involved researchers adopting an ‘embedded’ style of engagement to gain insider knowledge of organisational systems and to build trust. Researchers have spent time attending management meetings, strategic board meetings and training sessions across a variety of provider organisations ranging from clinical commissioning groups, hospital trusts, mixed stakeholder groups forming integration boards, social care and voluntary sector organisations. Co-design workshops have taken place to create evaluation and development strategies for service innovations. In one site, we have two researchers in residence who have been funded by the Torbay Medical Research Fund to investigate the ongoing process of integrating different services. In another site, a system-wide longitudinal formative evaluation has provided a rich picture of how services have developed P3C applying differential system leavers to support this process. Both sites have benefited from regular feedback to chart the development of the system and demonstrate where processes and outcomes are improving.

Figure 3 displays the geographical spread of the settings we are working with across the south west of England and Table 2 describes these settings, their models and the links that have been established or strengthened as a consequence of the collaboration.

**Table 2:** Examples of the service model innovations and organisational links

Name of service model	Description of service model	Links established as a result of the collaboration
Somerset Test and Learn	Roll out of the Symphony Complex Care model, developed in South Somerset, to other localities (Taunton and Mendips) across Somerset. Variations of linkage (networks) between primary, secondary and voluntary sector organisations.	South West Academic Health Science Network, Department of Community and Primary Care Research Group, Plymouth University, Health Connections Mendip (NGO), Village Agents (NGO), University of York, general practices across Somerset, Yeovil district hospital, Musgrove Park hospital, South West clinical support unit, Somerset County Council and Frome community hospital.
Somerset Practice Quality Scheme	General practitioners applying a ‘system lever’ (discretion from pay for performance schemes) to enable the development of the above innovations	South West Academic Health Science Network, Department of Community and Primary Care Research Group, Plymouth University, 55 general practices in Somerset, Somerset Clinical Commissioning Group, Somerset County Council, Somerset Partnership Trust and National Health Service England.
Torbay Integrated Care Organisation	The integration of acute and community services across five localities in South Devon. A range of around 30 service innovations and enabling functions are being rolled out in the new care model programme, including the deployment of Third Sector ‘Well-being Coordinators’, enhanced intermediate care, and multi-disciplinary health and well-being teams in locality hubs with primary care input	South Devon Trust, Clinical Commissioning Group, Devon North East West Clinical Commissioning Group, Devon Partnership Trust, Torbay Council, Devon County Council, Healthwatch Torbay, Healthwatch Devon, Torbay Community Development Trust, Teignbridge Community and Voluntary Services, Volunteering in Health/Totnes Caring, Age UK Torbay, GP practices in Coastal Locality, South West Academic Health Science Network, Department of Community and Primary Care Research Group, Plymouth University and Oxford University.
Integrated Personal Commissioning	Two demonstrator sites (Torbay and Cornwall) implementing a form of integrated personal budget that links services to personalised goals via a budget allocation. A range of statutory and non-statutory services are brokered to achieve a coordinated and personalised plan based on the preferences of the individual patient.	NHSE national team, South West Academic Health Science Network, Department of Community and Primary Care Research Group, Plymouth University, Torbay Carers, Torbay Community Development Trust, Kernow Clinical Commissioning Group, Age UK, Devon North East West Clinical Commissioning Group and Torbay Council.

**Fig. 5:**
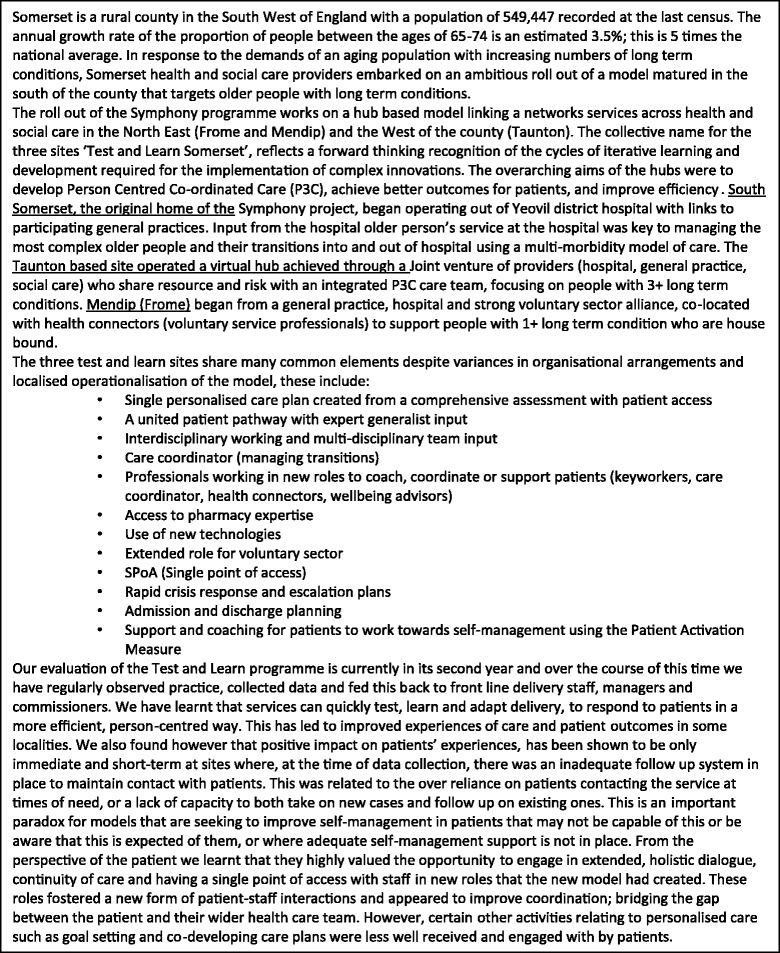
Somerset Test and Learn: the roll out and adaption of the south Somerset Symphony model

**Fig. 6:**
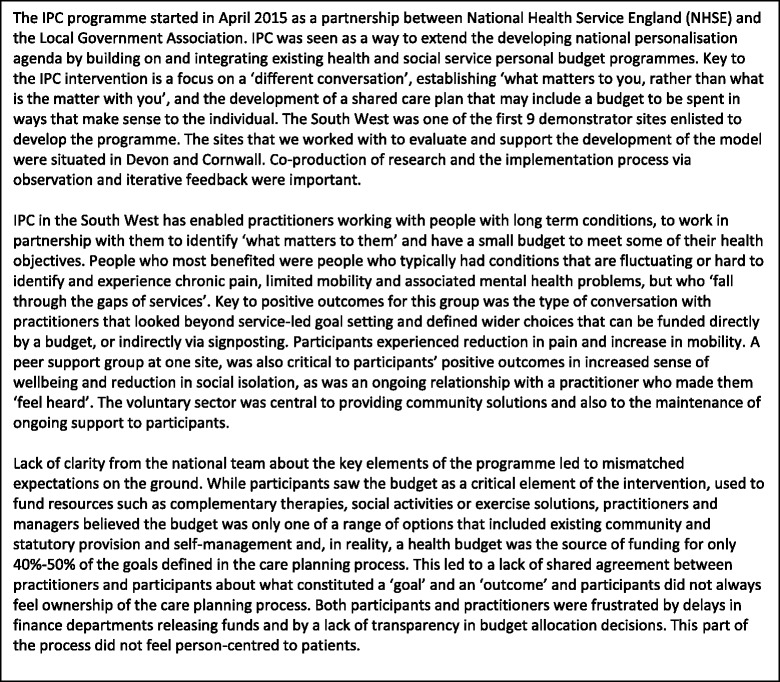
Local Implementation of Integrated Personal Commissioning (IPC) in the South West

The following two brief vignettes provide a more detailed snapshot of two of these service change models that we have been working with (Figs. 5 and 6).

While evaluation is, of course, crucial to establish what works well and what not so well within these new models of care, it is important to remember that these new models are attempting to change not only processes, but also practitioner and patient mindsets, within workplaces with highly ingrained cultures. Such changes take a substantial amount of time to embed. In addition, while it is important for models to learn and evolve, continued communication and some aspect of continuity is essential for service teams and patients if they are to feel engaged, important and part of a service.

## Discussion

Our experience, so far, has been positive; services want support and engagement and to create an evidence base for monitoring and reflecting on what changes they make. There have been challenges, and the pressure services are under, in the form of policy drivers and the high turnover of senior managers and frontline staff, are also barriers for us to overcome. However, sustained engagement from commissioners and a range of providers has helped to reduce the impact that these barriers have imposed upon our evaluations and the services being delivered. The collaboration between the southwest Academic Health Science Network and the SW CLAHRC has also been instrumental in supporting engagement and ensuring robust evaluation. This is due to the active and trusted links that both organisations have with commissioning and service delivery organisations across the region and a strategic commitment to work in partnership with the local health and social care sectors. However, this collaborative effort will only work with sustained support from commissioners and providers. By recognising and addressing the current problems in the system as a collaborative effort, progress towards a more efficient and person-centred healthcare system becomes possible. The level of change required is profound and we should not be afraid to highlight the scale of the problem and the probable solutions.

Anecdotal evidence is beginning to suggest that the process is influencing practice. However, working in this way is not without its challenges, and we are a long way off from the development of a linked data set locally or across the region that will allow us to assess impact innovations from a cost perspective. Trust is dependent on the exchange of services and interactions and this takes time to establish. We are beginning to see data flow and hope that this will continue in a constantly changing and open system.

At this stage, we need to acknowledge our state of uncertainty about which approaches to P3C are effective, whether it is possible to facilitate a sustained cultural change towards person-centred practice or whether this is context dependent. We are also currently unsure about whether the transactional costs required for coordinated care can be recouped by reductions in service costs and whether we can start to collect adequate data on the experience of care through everyday practice. It will be several years before we have enough data to bring together local evaluations and health economy metrics and dashboards for stakeholders and researchers to draw conclusions about what is effective.

## Conclusions

The current pressures on health and social care systems, with the expectations to deliver more efficient and person-centred models of care, now require a new modus operandi. We are developing and testing an approach based on collaborative action where research and innovative practice are brought together as partners to reduce uncertainty and provide timely practical knowledge and evidence to support those at the coalface delivering new models of P3C.

## Abbreviations

P3C: person-centred coordinated care
SW CLAHRC: South West Peninsula Collaboration for Leadership in Applied Health Research and Care
